# Odd-Leg Birdcages for Geometric Decoupling in Multinuclear Imaging and Spectroscopy

**DOI:** 10.1155/2023/7137889

**Published:** 2023-04-10

**Authors:** Joseph Busher, Edith Touchet-Valle, Chenhao Sun, Steven M. Wright, Mary P. McDougall

**Affiliations:** 1 Department of Biomedical Engineering, Texas A&M University, College Station, TX 77840, USA; 2 Department of Electrical and Computer Engineering, Texas A&M University, College Station, TX 77840, USA

## Abstract

The utility of interleaved odd-number leg birdcage coils is demonstrated for decoupling in double- and triple-tuned multinuclear applications. The birdcage was designed to geometrically decouple from a planar double-tuned (^1^H-^23^Na) array and from a ^31^P saddle coil insert to create a triple-tuned configuration. Comparisons between an actively detuned coil and a purely geometrically decoupled architecture were used to demonstrate the capabilities of the design. In particular cases, the simplicity and adaptability of the interleaved nine-leg design for multinuclear nuclear magnetic resonance (NMR) offer a straightforward alternative to the often complex and lossy designs currently available for multinuclear birdcages and volume coils.

## 1. Introduction

The development of multinuclear nuclear magnetic resonance (NMR) techniques yields many unique challenges for engineers in the clinical adoption of these methods. Sensitivity to X-nuclei such as ^23^Na, ^31^P, and ^13^C has the potential to elucidate underlying physiological processes for the study and treatment of numerous disease states [1–5]. These nuclei, however, have much lower natural abundance and sensitivity than hydrogen and thus require specialized hardware for detection, such as array coils to increase sensitivity and signal-to-noise ratio (SNR) [6, 7]. Additionally, achieving reliable shimming in X-nuclei studies is a challenge and immediately presents the need for double-tuning with ^1^H [8]. Given the low sensitivity when detecting naturally abundant second nuclei, the short lifetime, expense, and nonuniform distribution in hyperpolarized applications, shimming requires the use of a ^1^H coil without moving the X-nucleus coil between scans. This system of multinuclear transmit and receive hardware requires a complex chain of specialized coils, multiband amplifiers, receivers, and often switching drivers, either for switch-tuned coils or active detuning. The process of designing these multiband systems benefits from low-complexity coil designs to test individual components for the optimization of the resultant system, initially motivating the volume coil design presented here [9].

One of the most common transmit coil designs is the birdcage due to its highly homogeneous field over a large volume [10, 11]. Traditionally, birdcages are constructed of two end rings and integer multiples of four evenly spaced legs connecting these end rings to create the symmetry and respective highly homogeneous fields characteristic of birdcage coils [12]. To create multituned birdcages, one of the most common methods is to add traps on the rungs [13–15]. This design involves an iterative tuning process for multiple traps while maintaining the circular symmetry of the birdcage. The result is therefore highly effective, but can be complex and results in a reported loss of quality factor (*Q*) [16]. A four-ring design also has been proposed in which an inner birdcage is constructed between two outer birdcages using common legs but different end rings [17–19]. This configuration creates a homogeneous double resonant structure, but with a difference in filling factors potentially limiting its scalability in size for clinical imaging. Interleaved birdcages for multinuclear applications attempt to minimize the losses in *Q* and filling factor discrepancies associated with other configurations. This design has previously been reported at various field strengths for hydrogen, sodium, and phosphorus [20–22]. Using interleaved odd-number leg birdcages enables a simple construction method to simultaneously create parallel linear fields at both frequencies, thus allowing for the possibility of geometrically decoupling from planar receive elements or any additional coil with orthogonal sensitivity. Specifically, we report benchtop and imaging experimental data for interleaved nine-leg birdcages tuned to ^1^H and ^23^Na at 4.7 T designed to accommodate a planar double-tuned receive array. Additionally, we report benchtop characterizations for a ^31^P volume saddle coil insert as a triple-tuned volume coil to further demonstrate the adaptability of the modular coil design.

## 2. Materials and Methods

### 2.1. Coil Construction.

A total of four volume coils were constructed for ^1^H (200 MHz), ^23^Na (53 MHz), and ^31^P (81 MHz) at 4.7 T (Table 1): two single-tuned birdcages (^1^H, ^23^Na), one interleaved double-tuned birdcage (^1^H-^23^Na), and one saddle (^31^P). All three birdcage configurations were constructed of 1oz copper-clad FR4 mounted on a 17.8 cm diameter acrylic former. Two nine-leg single-tuned birdcages were constructed: a 24.2 cm long sodium coil in a low-pass configuration with two capacitor breaks along the legs for distributed capacitances (1111C, Passive Plus), and a 21 cm long single-tuned bandpass birdcage for hydrogen. To account for nuances in the nine-leg design compared to conventional birdcages, the modes of the low-pass birdcage were mapped using a crossed probe. The modes of a conventional eight-leg birdcage are well documented [23]. The nine-leg birdcage showed similar behavior with multiple modes, the lowest mode of which was the homogeneous mode, and several degenerate modes at higher frequencies. Similarly, the modes of the ^1^H bandpass birdcage were also mapped. A unique characteristic of the bandpass birdcage is the greater control over the frequency of the modes based on design specifics [24]. This was therefore an essential component of the tuning process to verify the frequencies of the homogeneous and degenerate modes. The multinuclear birdcage was constructed by interleaving the nine-leg hydrogen coil and the nine-leg sodium coil on a single 17.8 cm diameter former. Conductor overlaps between the coils were electrically isolated using Kapton^®^ tape, and feed ports on opposite sides of the coil created a double-tuned coil with parallel fields at the two frequencies. The coil was constructed by first coarsely tuning the birdcages using the same distributed capacitances as their single-tuned counterparts. The capacitances of the sodium coil were then adjusted on the legs on either side of the feed port to steer the fields to be precisely aligned, as verified by a field probe. All coils were matched and tuned with variable capacitors (NMAT 40HVE 1712, Voltronics Corp.). A shield was constructed for all three birdcages using overlapping sheets of 0.25 oz Pyralux^®^ copper-clad laminate on a 25.4 cm acrylic former.

**Figure 1:**
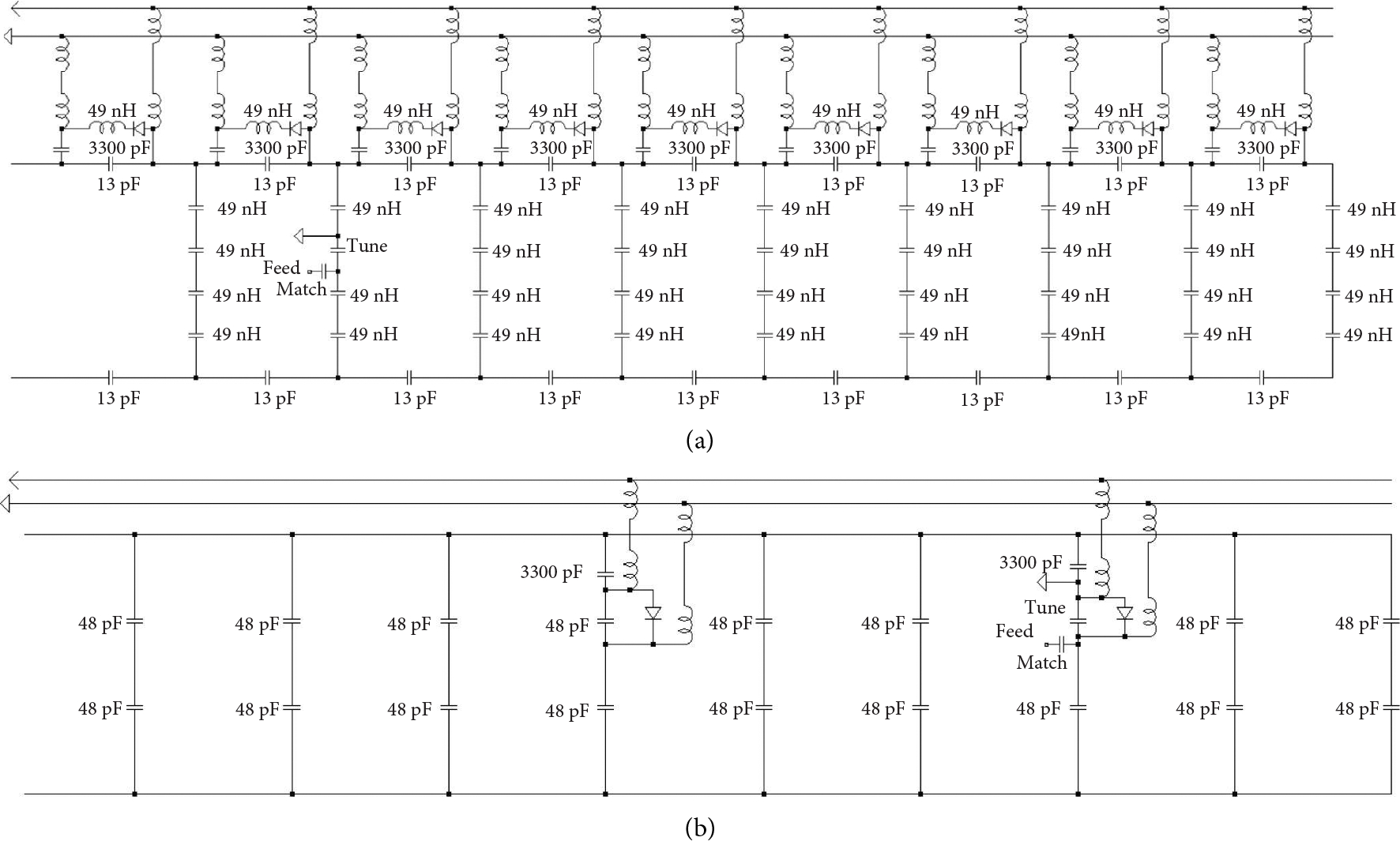
(a) Schematic for the hydrogen birdcage with active detuning circuitry. Unlabeled inductors are 6.8 *μ*H RF chokes. (b) Schematic for the sodium coil with active detuning schematic. Unlabeled inductors are 6.8 *μ*H RF chokes.

A standard active detuning network was added to the multinuclear birdcage to be able to compare/evaluate using geometric decoupling alone from a planar receive array to using active detuning [25]. A tuned active trap was added on each capacitor of one end ring of the ^1^H coil. The active trap was constructed using variable inductors (165 Series, Coil Craft) and active pin diodes (MA4P7006F-1072-T, MACOM). Alternatively, the sodium coil was detuned with a different network by adding an active PIN diode in parallel with the variable tune capacitor and a distributed capacitor on another leg 80 degrees offset from the feed port to fully detune the coil. This simpler network was shown to shift the resonant frequency of the coil to effectively detune the coil, given the larger distributed capacitors on the sodium birdcage as compared to the hydrogen coil. Schematics for the multinuclear coil with active detuning networks are shown for ^1^H in Figure 1(a) and ^23^Na in Figure 1(b). A custom PIN diode driver was designed to integrate the active detuning into the control logic of the scanner following the designs of White [26]. The driver was designed to output 1.6 A of current to detune the birdcage and 48 V to reverse bias during transmit.

**Figure 2:**
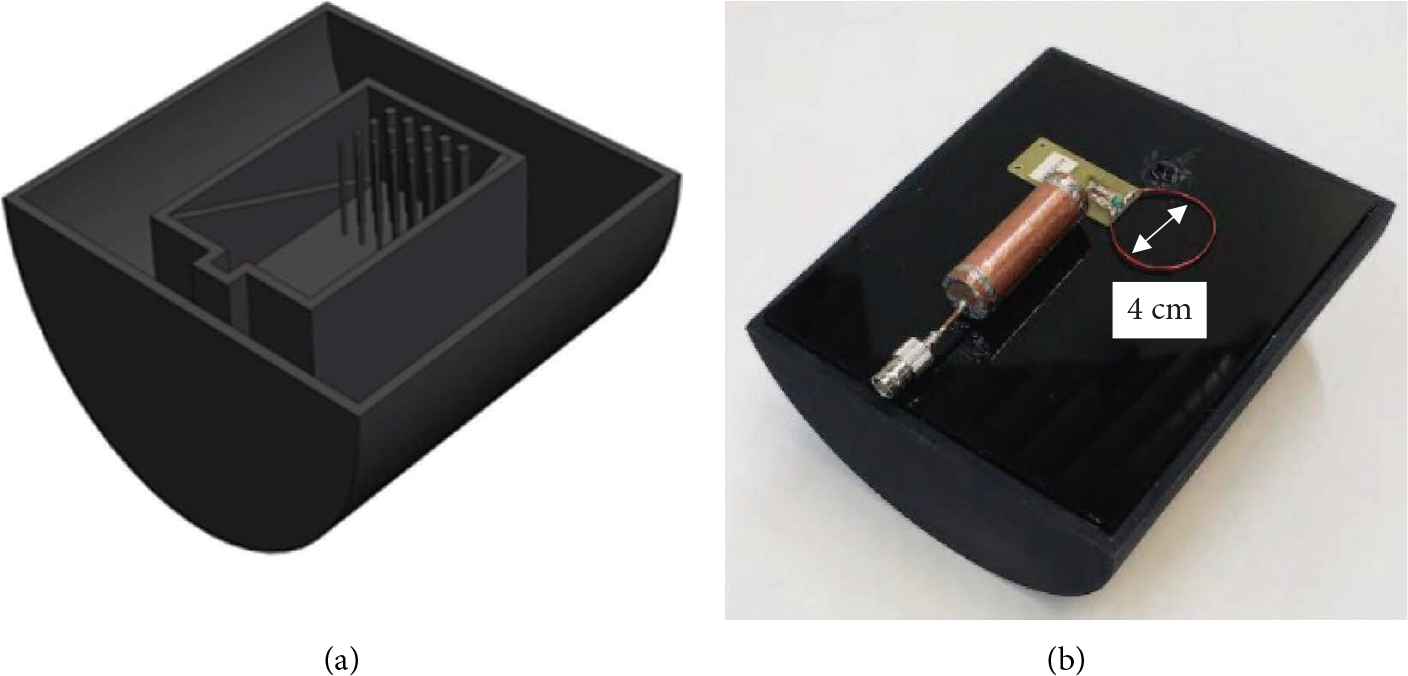
(a) CAD rendering of the phantom showing the inner structure with the rectangular flood region with the ramps and pin cushion design for spatial localization and resolution quantification, respectively. (b) Photograph of the phantom with a single receive loop coil positioned on top.

**Figure 3:**
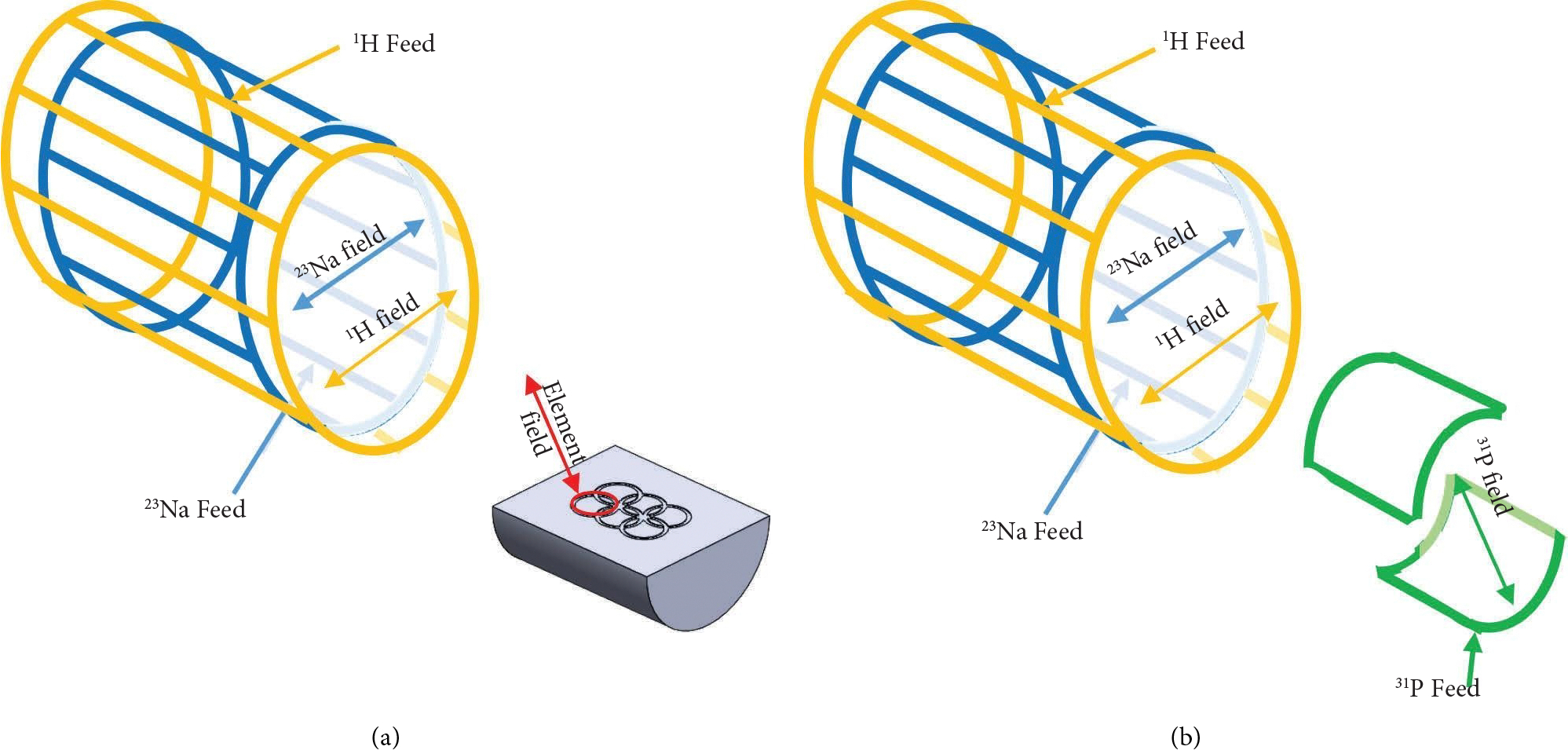
(a) Graphic showing the double-tuned birdcage net field directions and the orthogonal field orientation of the planar array element on the phantom including the feed locations. (b) Graphic showing the triple-tuned birdcage net field directions and the orthogonal field orientation of a saddle insert including the feed locations.

To test the ability of the birdcage to decouple from planar receive elements, two loops were constructed out of 24AWG wire with a 4 cm diameter, one for sodium (53 MHz) and one for hydrogen (200 MHz) matched and tuned with variable capacitors (SGC3S, Sprague Goodman). These loops were then moved into each of the six positions in the volume coil based on where they potentially would be located in a six-element array in order to test geometric decoupling and find the “worst-case scenario” for comparison to active detuning. A dedicated phantom former was designed (Figure 2(a)) and 3D printed out of polyamide (HP PA 12) on an MJF (Material Jet Fusion) printer. The former also provided a platform for positioning of the receive coils (Figure 2(b)) and to maintain the orthogonal fields (Figure 3(a)) between the loops and birdcages for a consistent decoupling angle. The phantom was composed of a single homogeneous flood region of NaCl (*σ* = 0.73 S/m, *ε*_*r*_ = 78). Ramps were added for slice localization and a pin cushion structure was added for quantifying resolution. The lid of the phantom was etched to accommodate a six-element receive array, or in this case, to indicate where to place the single loop to test coupling at the various relevant positions.

Finally, to further demonstrate the utility of the parallel fields generated by this architecture, a phosphorous saddle coil insert was constructed to create a triple-tuned volume coil. This coil was positioned within the birdcage such that it created net linear fields orthogonal to the net birdcage fields, resulting in a purely geometrically decoupled triple-tuned volume coil (Figure 3(b)). The saddle coil was created using adhesive copper tape on a 12.7 cm diameter acrylic tube with a length of 17 cm. The saddle coil was then coarsely tuned with distributed capacitors (1111C, Passive Plus) and finely matched and tuned with variable capacitors (NMAT 40HVE, Voltronics). Bench measurement characterizations were performed to demonstrate the matching and decoupling capabilities of the saddle from the birdcage.

### 2.2. Benchtop Measurements.

Bench measurements were used to characterize coil performance and the decoupling abilities of the different coil configurations presented here. All bench measurements were taken on a vector network analyzer (E5071C, Agilent). *S*_11_ and *Q* were measured and compared between the single-tuned birdcages and double-tuned birdcage configurations with and without active detuning. Active detuning was verified using a crossed probe to show no resonant peak at the frequencies of interest. An *S*_21_ measurement was used to note any coupling between the two birdcage ports, and a field probe was used to map the homogeneity of the birdcage using an in-house field mapping system. The decoupling between the birdcage and loop elements in the six different locations was assessed with an *S*_21_ measurement taken between the birdcage port and the loop. Although this measurement does not define absolute sufficient decoupling given the large differences in coil sensitivity between the large transmit birdcage and the smaller receive loops, it allowed for quantification and comparison of decoupling at different positions within the birdcage. In this way, the worst case was found for the fairest comparison to active detuning. Similarly, *S*_11_ and *S*_21_ measurements were acquired with the ^31^P saddle coil to verify sensitivity and coupling measurements on the bench.

### 2.3. Imaging.

All imaging was performed on a 4.7 T Varian Inova scanner. The single receive loop was positioned in the location of the array determined by *S*_21_ measurements to simulate the worst-case coupling scenario in a receive array configuration. Images were acquired using the birdcage in transmit-receive mode for ^1^H to verify homogeneity and to note any distortions potentially due to coupling to the receive loop. A standard spin echo pulse sequence was used with: TR/TE: 1000 ms/30 ms, m: 256 × 256, FOV: 160 mm × 160 mm, and slice thickness: 1 mm. The same pulse sequence was then run while receiving with the ^1^H loop both with and without active detuning to compare the effects. Sodium images were acquired with and without active detuning using a standard gradient echo sequence: TR/TE: 30 ms/3.5 ms, N: 64 averages, m: 64 × 64, FOV: 160 mm × 160 mm, slice thickness: 20 mm, and spectral width: 20 kHz. Due to the low sensitivity, sodium images were only acquired using the loop to receive.

## 3. Results and Discussion

### 3.1. Volume Coil Construction.

**Figure 4:**
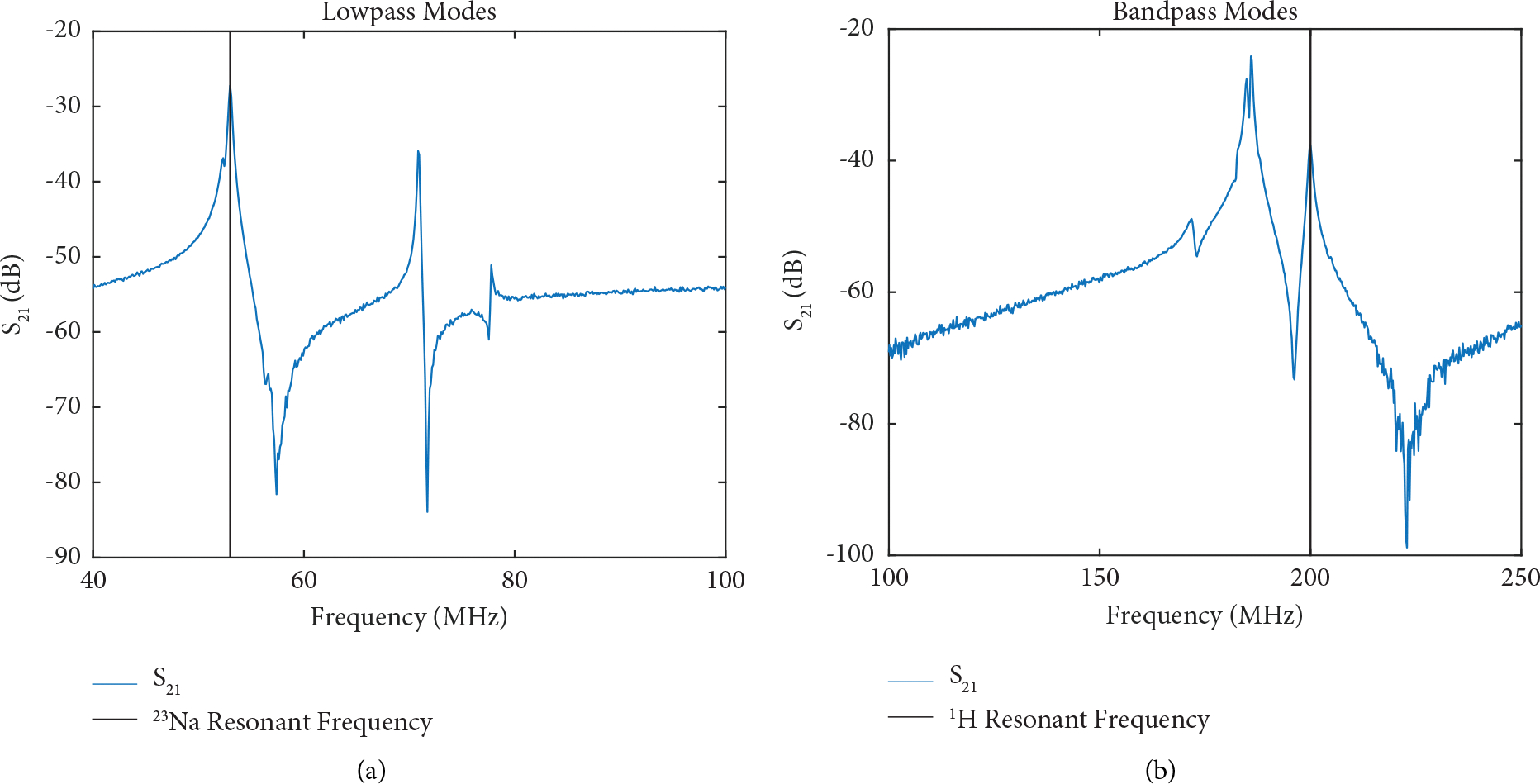
Modes detected by a crossed probe centered in the coil: (a) Resonant modes of a single-tuned nine-leg lowpass birdcage designed for ^23^Na at 4.7 T. (b) Resonant modes of a single-tuned nine-leg bandpass birdcage designed for ^1^H at 4.7 T.

**Figure 5:**
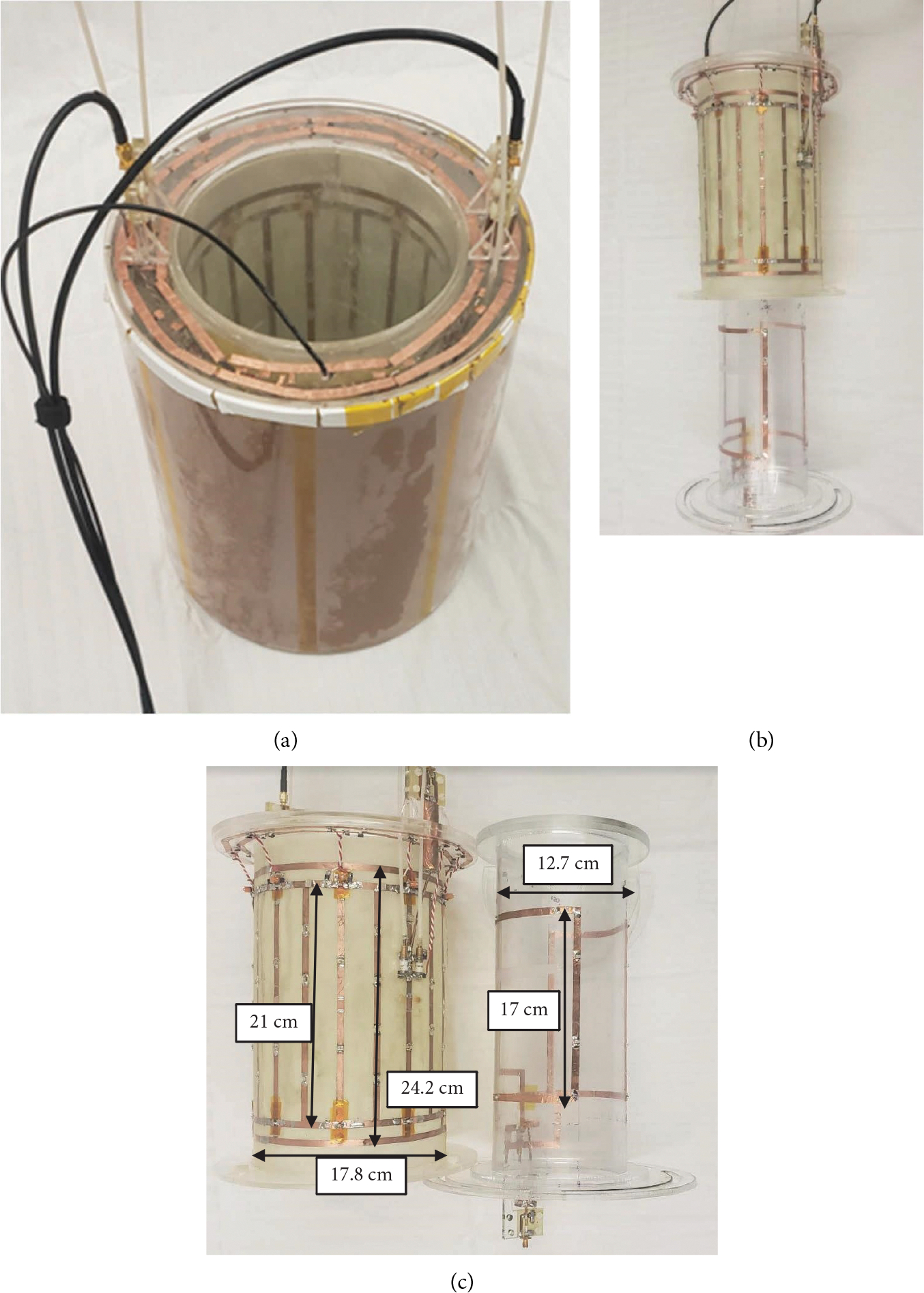
(a) Photograph of the double-tuned birdcage in the shield. (b) Photograph of the saddle coil insert into the double-tuned birdcage former. (c) Photograph of the saddle and birdcage side by side showing the dimensions and positioning of the saddle within the birdcage. Yellow Kapton^®^ tape is also visible, electrically isolating the hydrogen birdcage end rings from the sodium birdcage legs.

The interleaved coil design allowed for a straightforward decoupling mechanism to create aligned fields without the complex and lossy limitations typical of other double-tuned designs. The main modes of the single-tuned nine-leg lowpass (Figure 4(a)) and bandpass (Figure 4(b)) birdcages are shown with the homogeneous birdcage mode, and the main degenerate modes are measured with a single probe centered in the coil. The final double-tuned coil is shown in the shield in Figure 5(a). The concentric saddle insert is shown partially inserted in Figure 5(b), and side-by-side with dimensions to show relative longitudinal coil positioning in Figure 5(c).

### 3.2. Benchtop Measurements.

**Figure 6:**
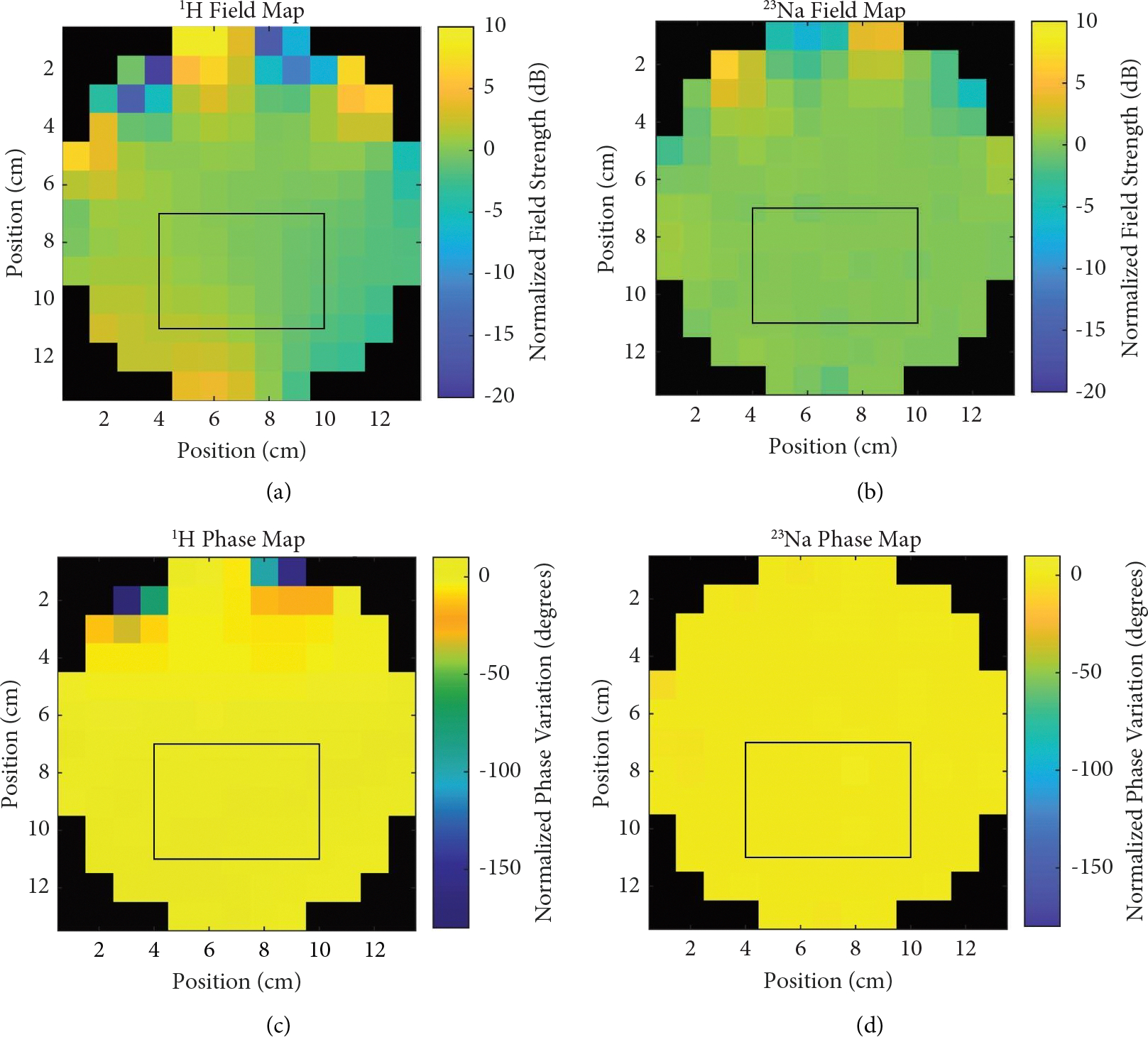
Double-tuned field maps (a) Axial field map for ^1^H coil normalized to show field variation with a black box highlighting the flood region of the phantom. (b) Axial field map for sodium coil normalized to show field variation with black box highlighting the flood region of the phantom. (c) Axial phase map of the ^1^H coil normalized to show phase variation in degrees from the center of the coil with black box highlighting the flood region of the phantom. (d) Axial phase map of the sodium coil normalized to show phase variation in degrees from the center of the coil with black box highlighting the flood region of the phantom.

Benchtop characterizations of the coil shown in Table 2 summarize the sensitivity and decoupling behavior of the birdcage with loaded measurements. While all coil configurations were shown to be well matched (better than −29.7 dB), the double-tuned configurations did show a 5% loss in *Q* for ^1^H and 3% for sodium when compared to the single-tuned designs. This loss in *Q* was compounded by the addition of active detuning circuitry, resulting in a 32% loss for ^1^H and 16% for sodium, thus significantly reducing transmit coil efficiency. *Q* ratios were taken for the single-tuned coil configurations and were shown to be 1.67 for ^1^H and 1.35 for ^23^Na. Additionally, coupling between the two birdcage ports was shown to be 4.6 dB worse at 200 MHz with the addition of active detuning suggesting some coupling through the detuning circuitry itself at that frequency. Axial field maps for the ^1^H coil (Figure 6(a)) and sodium coil (Figure 6(b)) show the homogeneity of the coil to be within 1.5 dB of variation over the flood region of the phantom with phase maps shown for ^1^H (Figure 6(c)) and sodium (Figure 6(d)).

Similarly, the triple-tuned volume coil configuration further demonstrated the geometric decoupling benefits of the interleaved birdcage design. The matching and tuning data (Table 4) demonstrated that all coil configurations could be easily matched to better than −21.7 dB. Quantifications of decoupling between the birdcage and saddle insert (Table 5) showed that port-to-port coupling was no worse than −14.6 dB and up to −30.2 dB depending on the frequency. This coil architecture benefits from the low-complexity approach of changing coil inserts to add or remove sensitivity to specific (or additional) nuclei and adjusting the coil to suit the required application. This particular nine-leg interleaved birdcage design was originally intended to be used as a double-tuned ^1^H-^23^Na transmit coil with planar receive elements, and the ^31^P insert coil was added to further demonstrate the utility of the parallel linear fields [27, 28]. This design could (and should) easily be modified for a given study to optimize sensitivity by arranging the coils such that the lowest-sensitivity nuclei are the insert, making it the most sensitive to the sample. It is worth noting that odd-legged birdcages cannot straightforwardly operate in a standard quadrature mode with conventional 90-degree-separated ports. The least complicated approach to making a single nucleus quadrature (presumably the less sensitive non-^1^H one) is the addition of an insert coil positioned orthogonally within the main coil. For example, in this case, the third-nucleus saddle coil could instead be tuned to the less sensitive ^23^Na frequency to provide quadrature operation. In summary, the straightforward decoupling and flexibility/modularity provided by the design are significant benefits of odd-leg birdcages.

### 3.3. Imaging Studies.

**Figure 7:**
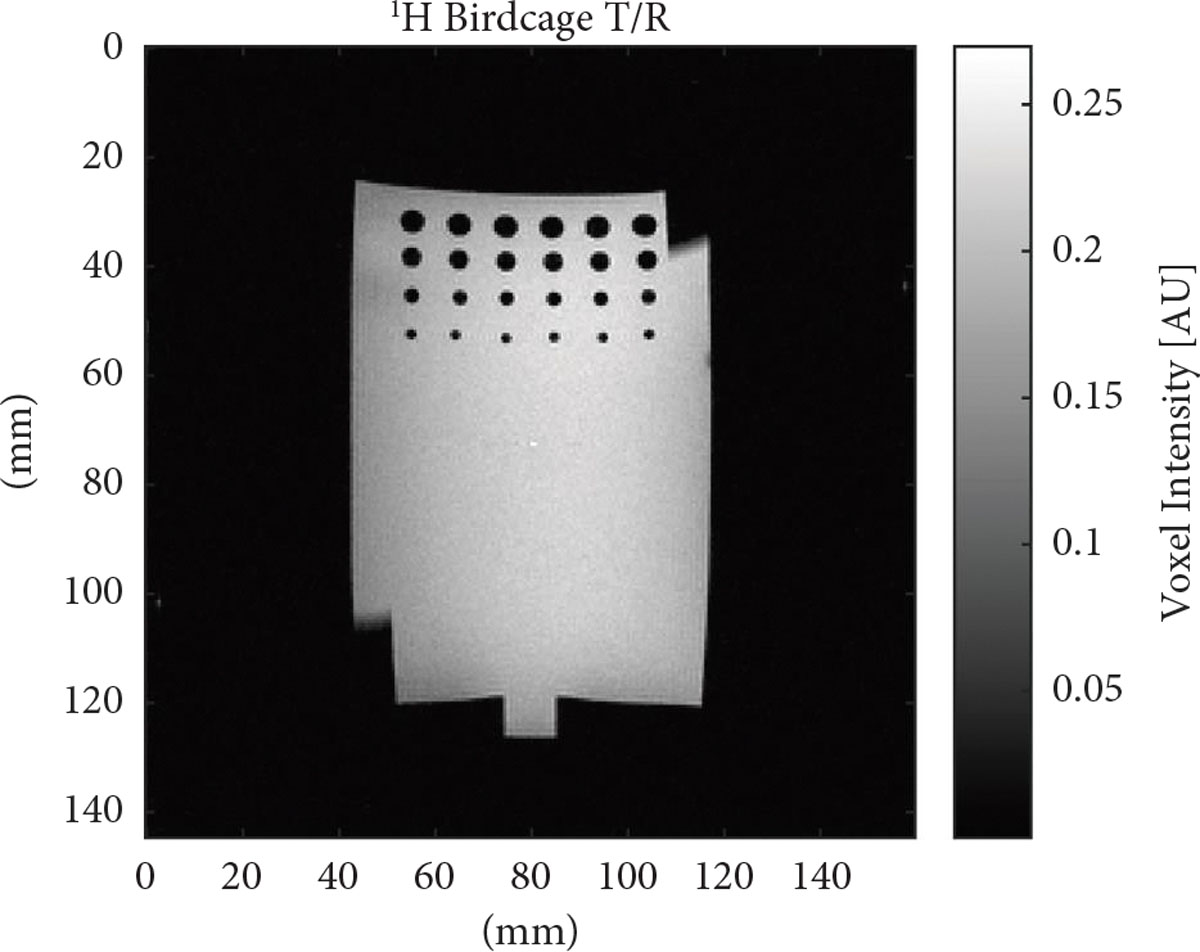
Double-tuned birdcage T/R image showing field homogeneity with a receive loop present but unused in the bottom left corner.

To demonstrate the homogeneity of the birdcage an image was first acquired using the birdcage in the T/R mode (Figure 7). This showed no visible distortions to the homogeneity of the field with a loop in place, suggesting sufficient decoupling during transmit between the birdcage and the receive array due simply to geometric decoupling. Due to the low sensitivity of the sodium birdcage, a T/R image was only acquired for ^1^H. However, given the similar sizes of the coils and comparable benchtop coupling measurements and field maps, it is expected that the same effect would be seen for sodium.

**Figure 8:**
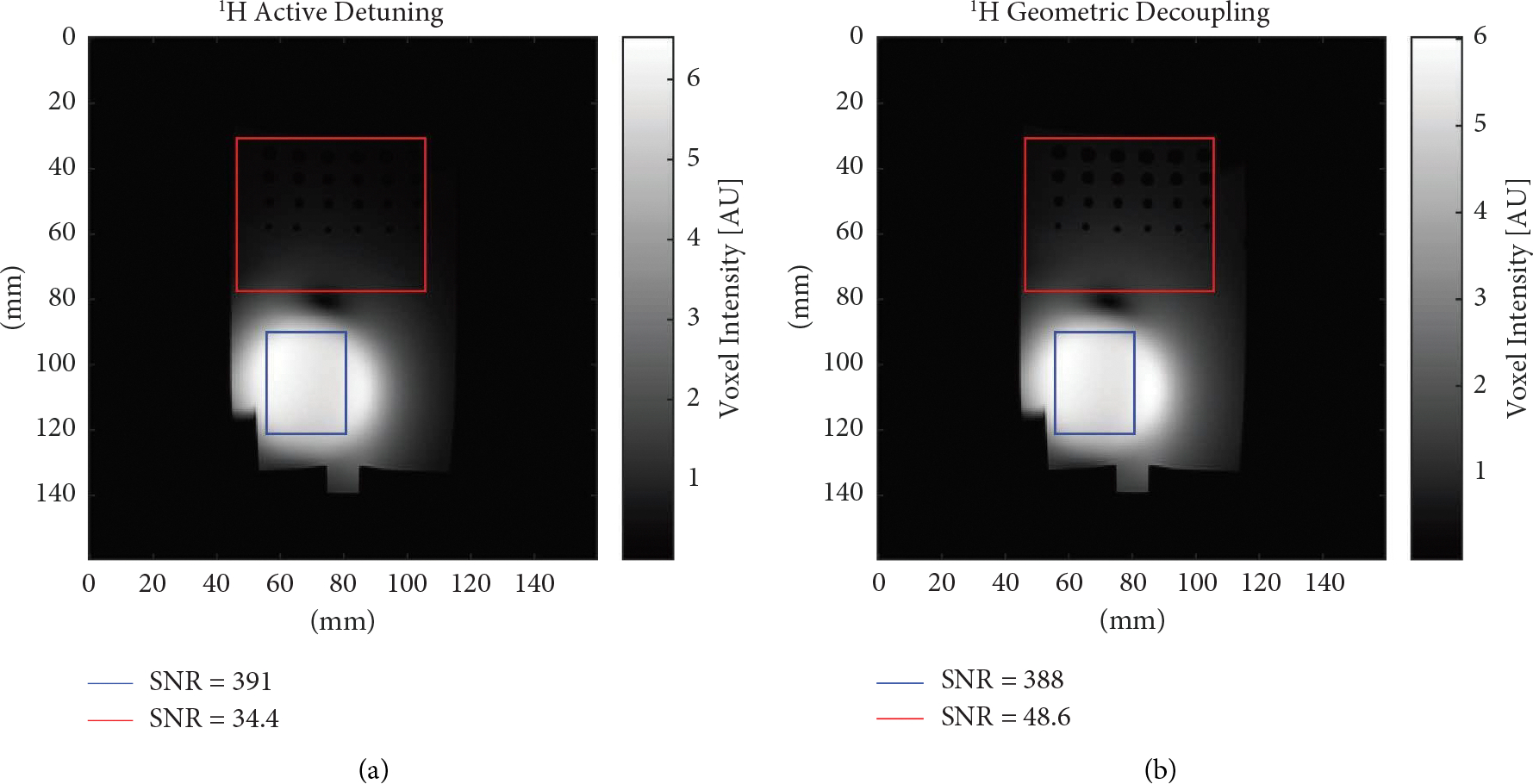
Images using the double-tuned birdcage with receive loop. (a) ^1^H image receiving with a single loop using active detuning. (b) ^1^H image receiving with a single loop using purely geometric decoupling.

^1^H images were acquired both with (Figure 8(a)) and without (Figure 8(b)) active detuning to demonstrate the effects of the additional decoupling of the active detuning circuitry. The average SNR was quantified for two regions as shown in Figure 8: a region highlighted in a blue box directly under the loop and a region highlighted in a red box within the phantom but not directly below the loop. The average SNR of these two regions was calculated by taking the average intensity of the regions within the two boxes and the average intensity of the same regions in a separate noise image acquired without the transmit power amplifier active. For consistency, SNR calculations were scripted to take the exact same regions in all four images (with and without active detuning at both nuclei). Raw SNR data is available in the supplementary table (available here) to show the influence of active detuning on both the average signal and noise levels in these regions. The region within the blue box was found to have an average SNR of 391 with active detuning compared to 388 with purely geometric decoupling. Similarly, the average SNR of the region within the red box was found to be 34.4 with active detuning compared to 48.6 with purely geometric decoupling. This suggests that roughly 41% more signal was detected with purely geometric decoupling compared to an actively detuned network in this region away from the loop, presumably through coupling between the birdcage and receive loop.

The coupling between the loop coils and birdcage (Table 3) was no worse than −28 dB with a range of 1.4 dB for sodium. The ^1^H coils coupled no worse than −26.5 dB with a range of 4.7 dB. Given that the variation was more significant for the ^1^H frequency and the higher sensitivity of this nuclei, the element in position six was used for imaging studies to demonstrate the worst-case coupling to validate the purely geometric decoupling design.

**Figure 9:**
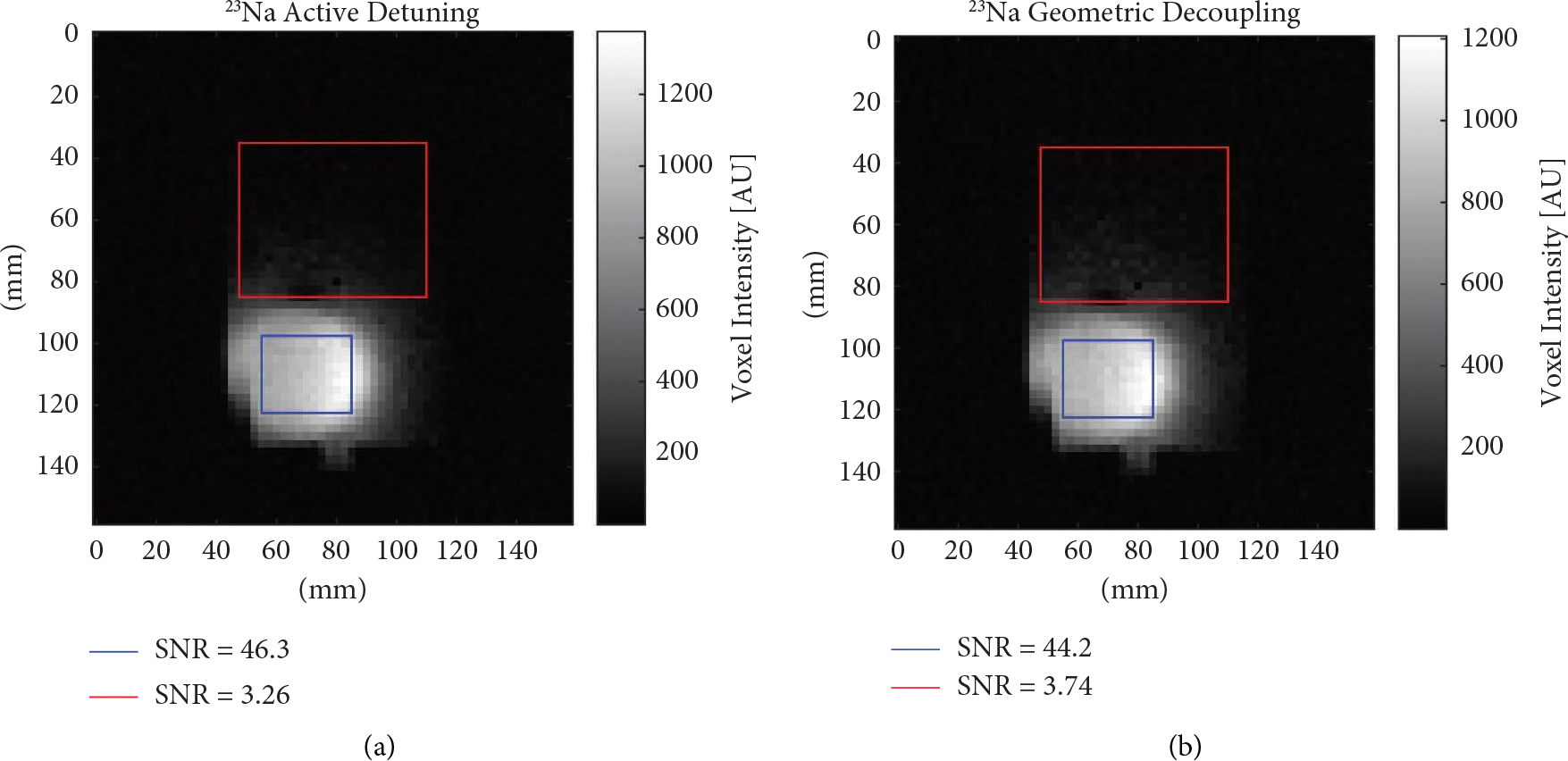
Images using the double-tuned birdcage with receive loop. (a): Sodium image receiving with a single loop using active detuning. (b): Sodium image receiving with a single loop using purely geometric decoupling.

Similarly, images were taken for sodium using the same experimental setup with and without active detuning. Given the lower sensitivity of these coils the background signal was not as visible either with (Figure 9(a)) or without (Figure 9(b)) active detuning. Quantifications of average SNR were made using the same regions of the phantom as with ^1^H with a region highlighted with a blue box directly under the loop and a region highlighted with a red box within the phantom but not directly below the loop. Directly under the loop, the average SNR was found to be 46.3 with active detuning versus 44.2 without. Outside the loop, the average SNR was found to be 3.26 with active detuning compared to 3.74 without, a roughly 15% increase in signal with purely geometric decoupling as compared to active detuning.

While imaging experiments did show active detuning leads to a decrease in coupling, the application must be considered in the decision of whether the added complexity and loss are needed. Certain applications of spectroscopy for low-sensitivity X-nuclei using receive element reception and requiring the absolute highest SNR achievable or those requiring the use of nonplanar arrays will make purely geometric decoupling as described inappropriate. Other applications where a slight reduction in SNR using a receive element is acceptable and will still yield the necessary results or where the coil will be used in the volume T/R configuration, this simple and adaptable hardware may be sufficient. In the end, the needs of a given study must be taken into consideration when designing the appropriate hardware.

## 4. Conclusions

Odd-numbered leg interleaved birdcages can generate parallel fields at two frequencies, creating a straightforward geometric decoupling mechanism from either planar receive elements or any additional insert with an orthogonal field. This was demonstrated with a set of nine-leg birdcages tuned to ^1^H and ^23^Na and reception loops at both frequencies, and the addition of a ^31^P saddle coil insert to create a triple-tuned coil. While the application of a purely geometrically detuned coil is limited to planar arrays or a T/R volume coil, a simple active detuning network can be easily added if the application requires further decoupling either due to geometric constraints or if the geometric decoupling is deemed insufficient for a given experiment. Given the simplicity and adaptability of the interleaved nine-leg design for multinuclear NMR spectroscopy and the additional decoupling provided by the geometric decoupling even with the addition of active detuning, this design offers a straightforward alternative to the many complex and lossy designs currently available for multinuclear birdcages and volume coils.

## Supplementary Material

Supplementary Table

A supplementary table has been included showing the raw SNR data used to calculate the average SNR values presented in Figures 8 and 9. (Supplementary Materials)

## Figures and Tables

**Table 1:** Coil construction configurations.

Nuclei	Configuration	Number of rungs	Diameter (cm)	Length	Shielded

Single-tuned ^1^H	Bandpass birdcage	9	17.8	21 cm	Yes
Single-tuned ^23^Na	Lowpass birdcage	9	17.8	24 cm	Yes
Double-tuned ^1^H/^23^Na	Nested birdcages: ^1^H bandpass/^23^Na lowpass	9 for each nucleus (18 total)	17.8	21 cm ^1^H 24 cm ^23^Na	Yes
Single-tuned ^31^P	Saddle coil	N/A	12.7	17 cm	Yes

**Table 2:** Double-tuned birdcage bench measurements.

Measurement	^1^H_Single-Tuned_	^23^Na_Single-Tuned_	^1^H_Double-Tuned_	^23^Na_Double-Tuned_	^1^H_Double-Tuned Active Detuning_	^23^Na_Double-Tuned Active Detuning_

*Q* (AU)	344	337	301	326	233	284
*S*_11_ (dB)	−29.7	−34.4	−41.6	−30.7	−37.5	−32.0
*S*_21_ (dB)	N/A	N/A	−21.2	−18.3	−16.6	−22.3

**Table 3:** Double-tuned birdcage-surface coil decoupling measurements.

Element position	^23^Na (dB)	^1^H (dB)

1	−29.4	−27.8
2	−29.2	−29.6
3	−28.0	−29.7
4	−28.4	−29.8
5	−28.7	−31.2
6	−29.4	−26.5

**Table 4:** Triple-tuned birdcage/saddle pair matching/tuning measurements.

Port	^*S*^_11 Triple-Tuned_ (dB)	^*S*^_11 Single-Tuned_ (dB)

^1^H	−25.1	−29.7
^23^Na	−24.6	−34.4
^31^P	−21.7	−27

**Table 5:** Triple-tuned birdcage/saddle decoupling measurements.

	*S*_21_ 53 MHz (dB)	*S*_21_ 81 MHz (dB)	*S*_21_ 200 MHz (dB)

^1^H-^31^P port	N/A	−30.2	−15.4
^1^H-^23^Na port	−18.5	N/A	−15.6
^31^P-^23^Na port	−14.6	−22.1	N/A

## Data Availability

The data used to support the findings of this study are included within the article.
