# Supplementary figures and images for: Odd-Leg Birdcages for Geometric Decoupling in Multinuclear Imaging and Spectroscopy
Source: Concepts Magn Reson Part B Magn Reson Eng. Author manuscript; Available in PMC 2025 Oct 14. (PMC12517185; doi:10.1155/2023/7137889)

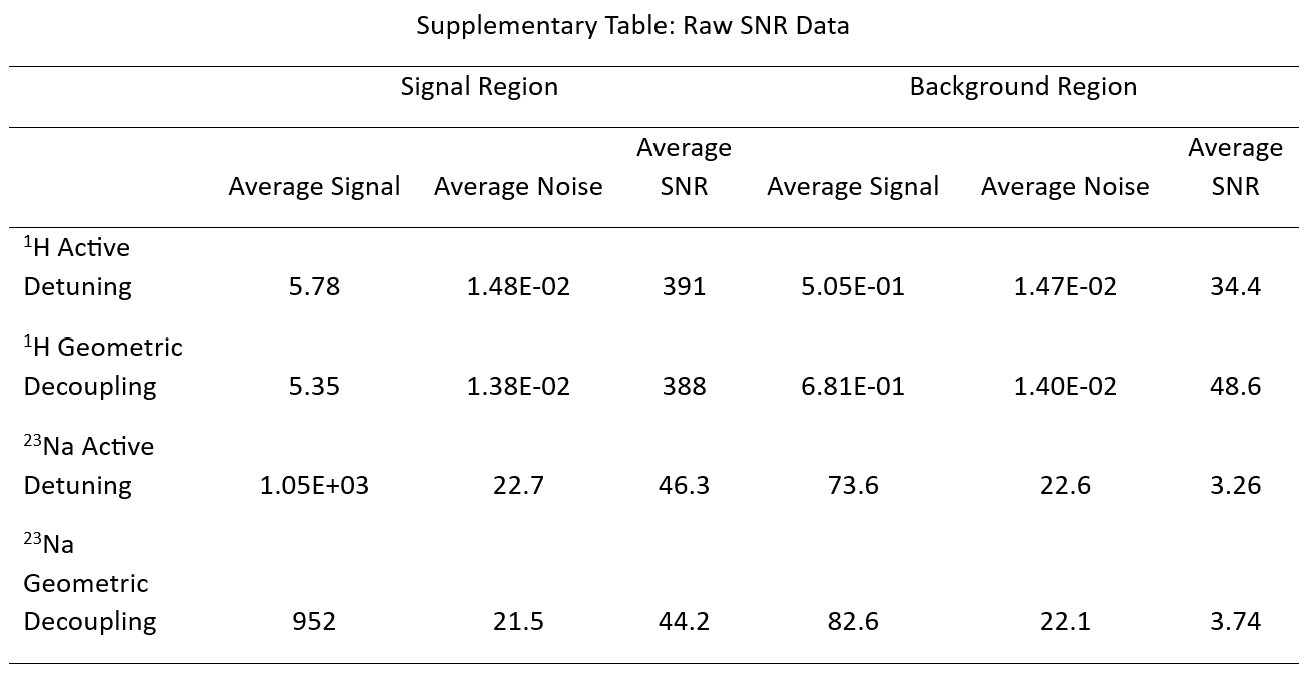

Supplement: Supplementary Table [file NIHMS2110857-supplement-Supplementary_Table.png]
